# Specific Features for the Competent Binding of Substrates at the FMN Adenylyltransferase Site of FAD Synthase from *Corynebacterium ammoniagenes*

**DOI:** 10.3390/ijms20205083

**Published:** 2019-10-14

**Authors:** Sonia Arilla-Luna, Ana Serrano, Milagros Medina

**Affiliations:** 1 Department of Biochemistry and Molecular and Cellular Biology, Faculty of Sciences, and Institute of Biocomputation and Physics of Complex Systems (Joint Units: BIFI-IQFR and GBsC-CSIC), University of Zaragoza, 50009 Zaragoza, Spain; 2 Centro de Investigaciones Biológicas, CSIC, Ramiro de Maeztu 9, E-28040 Madrid, Spain

**Keywords:** prokaryotic FAD synthase, species-specific traits, adenylyltransferase activity, head-to-tail organization, isothermal titration calorimetry, site-directed mutagenesis, biosynthesis, steady-state kinetics

## Abstract

Bifunctional FAD synthases (FADSs) catalyze FMN (flavin mononucleotide) and FAD (flavinadenine dinucleotide) biosynthesis at their C-riboflavin kinase (RFK) and N-FMN:adenylyltransferase (FMNAT) modules, respectively. Biophysical properties and requirements for their FMNAT activity differ among species. Here, we evaluate the relevance of the integrity of the binding site of the isoalloxazine of flavinic substrates for FMNAT catalysis in *Corynebacterium ammoniagenes* FADS (*Ca*FADS). We have substituted P56 and P58, belonging to a conserved motif, as well as L98. These residues shape the isoalloxazine FMNAT site, although they are not expected to directly contact it. All substitutions override enzyme ability to transform substrates at the FMNAT site, although most variants are able to bind them. Spectroscopic properties and thermodynamic parameters for the binding of ligands indicate that mutations alter their interaction modes. Substitutions also modulate binding and kinetic properties at the RFK site, evidencing the crosstalk of different protomers within *Ca*FADS assemblies during catalysis. In conclusion, despite the FMNAT site for the binding of substrates in *Ca*FADS appearing as a wide open cavity, it is finely tuned to provide the competent binding conformation of substrates. In particular, P56, P58 and L98 shape the isoalloxazine site to place the FMN- and FAD-reacting phosphates in optimal geometry for catalysis.

## 1. Introduction

**Figure 1:**
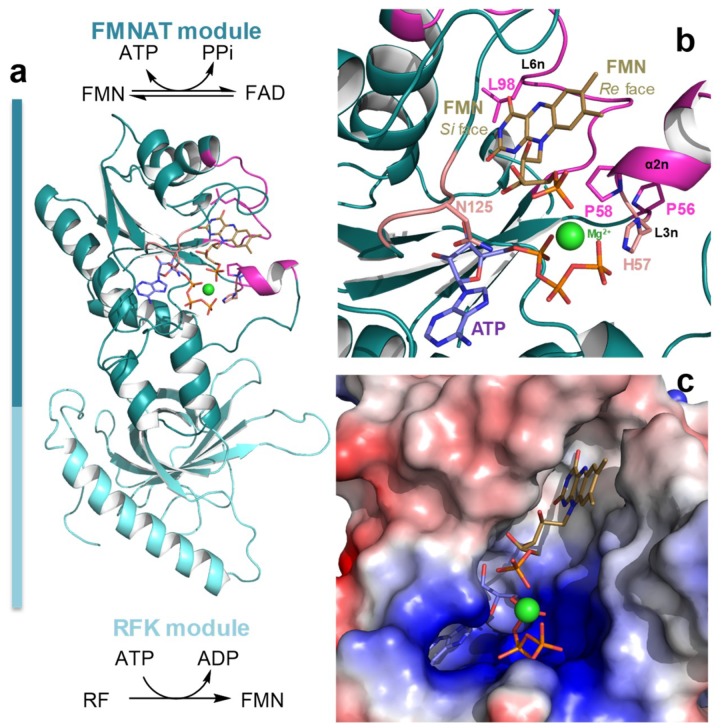
The three-dimensional structure of *Ca*FADS. (**a**) Cartoon representation of the overall folding of *Ca*FADS (PDB code: 2x0k) [3] and (**b**) details of substrates theoretically docked at its binding N-FMN:adenylyltransferase (FMNAT) active site according to [20]. Side chains evaluated in the present study (P56, P58 and L98) are shown in Corey, Pauling, Koltun (CPK) sticks with C in magenta. Structural elements containing them, L3n, α2n and L6n (n denoting secondary structures in the FMNAT N-terminal domain) and sited at the FMN *Re* face are also highlighted in magenta. Side chains of N125 and H57 are in CPK sticks with C in salmon. (**c**) Details of the FMNAT active site surface electrostatic potential with the docked substrates. In all panels, docked FMN and ATP substrates at the FMNAT module are shown as CPK sticks with C atoms in brown and violet, respectively, and Mg^2+^ is shown as a green sphere.

Prokaryotic FAD synthases (FADSs) are bifunctional and bimodular enzymes that catalyze the transformation of riboflavin (Vitamin B_2_, RF) into flavin adenine dinucleotide (FAD) through two sequential activities; first, an ATP:riboflavin kinase activity (RFK, EC 2.7.1.26) phosphorylates the RF substrate to produce flavin mononucleotide (FMN) and, then, an ATP:FMN:adenylyltransferase activity (FMNAT, EC 2.7.7.2) adenylates FMN to yield FAD, with this last reaction being reversible (Figure 1) [1]. By contrast, these two activities are split in two different proteins in most eukaryotes, particularly in mammals. The C-terminal module of the prokaryotic FADS family is responsible for the RFK activity and shares sequence and structural homology with monofunctional eukaryotic RFKs [2,3,4].

However, the N-terminal module, holding the FMNAT and FAD pyrophosphorylase (FADpp) activities, widely differs in structure and chemistry from eukaryotic enzymes catalyzing FAD biosynthesis [3,5,6,7,8,9]. Instead, it belongs to a structural superfamily (nucleotidyltransferase (NT)) that catalyzes the transfer of nucleotidyl groups to a broad range of substrates. The lack of homology between prokaryotic and eukaryotic FADSs has pointed to the FMNAT modules of prokaryotic FADSs as interesting antimicrobial targets [1,10] because interrupting its FMNAT activity in pathogenic bacteria would not affect FAD biosynthesis in the eukaryote hosts. In this line, the FMNAT module of bacterial FADSs is already being used as a target in the discovery of antimicrobials [10].

The FADS from *Corynebacterium ammoniagenes* (*Ca*FADS) has been extensively studied as model of the prokaryotic FADS family [3,11,12,13,14,15]. Nonetheless, recent studies using other family members, such as the FADSs from *Streptococcus pneumoniae* (*Sp*FADS) and *Listeria monocytogenes* (*Lm*FADS), envisage species-specific traits that modulate their efficiency. The reported differences among prokaryotic FADSs include (i) the stabilization or not of quaternary organizations during catalysis, (ii) the differential modulation of the RFK activity by substrates and products, (iii) the influence of the redox environment in the occurrence and/or efficiency of the RFK and/or FMNAT activities, and (iv) cooperation in the binding of substrates and products [14,16,17,18,19,20]. These data exemplify alternative strategies for FMN and FAD biosynthesis and homeostasis in different bacteria and, in some of them, they point to the FMNAT activity as a “redox sensor” of the media. These features increase the interest of prokaryotic FADSs in the discovery of species-specific antimicrobials. Nonetheless, before such applicability is achieved, further knowledge of differential traits in proteins from different species is required, particularly at the FMNAT activity.

No experimental structures have been reported so far for the FMNAT module of any prokaryotic FADS in complex with substrates or products. However, models for the interaction of *Ca*FADS with its ATP:Mg^2+^ and FMN substrates have been built; first, manually, but, more recently, optimized by docking and molecular dynamics (MD) simulations [3,20]. Together with site-directed mutational studies on conserved motifs in both the FADS and NT families, these models have pointed to several residues on the FMNAT module of *Ca*FADS as responsible for the stabilization of ATP and for the transfer of its nucleotidyl group to FMN [12]. In addition, variations in residues both coating the flavinic binding site and the channel between the ATP:Mg^2+^ and FMN binding sites at the FMNAT module have been claimed as responsible for the variability in requirements for FAD biosynthesis among prokaryotic proteins from different species [16,17]. 

In the present study, we evaluate the relevance for catalysis of the native integrity of the binding cavity for the isoalloxazine of the FMN and FAD substrates in the FMNAT module of *Ca*FADS (Figure 1) [3,20]. The three residues mutated here—P56, P58 and L98—are in general conserved in FADSs but not in other NTs, suggesting that they are somehow involved in the interaction with the FADSs specific substrate, the flavinic substrate. In fact, they are at the binding cavity of the flavinic substrate isoalloxazine moiety, although according to our molecular models they are not predicted to directly contact it (Figure 1b). We have produced the single variants P56W, P58W, L98W, L98K and L98A and the double variant P56A/P58A. These mutations introduce aromaticity and increase the volume in the corresponding residue (Trp), alter the residue nature (Lys) or include secondary structure flexibility (the replacement of Pro by Ala). The characterization of these variants indicates that, in *Ca*FADS, the FMNAT flavin cavity is carefully designed to position the isoalloxazine ring in such a way that the reacting phosphate atoms of the FMN or FAD substrate situate in the adequate geometry for the catalysis to take place. These observations also point to the discovery of compounds able to specifically bind at the isoalloxazine pocket of selected bacterial FADSs as a strategy to develop new potential antimicrobials.

## 2. Results 

### 2.1. Occurrence of P56, P58, and L98 Residues in Different Prokaryotic FADSs 

Residues P56, P58, and L98, situated at the FMNAT site of *Ca*FADS, are highly conserved in the prokaryotic FADS family [21]. The two prolines are part of a –FxphP- consensus region (highly conserver residues are in capital letters, x denotes any residue) (Figure S1a). P58 is conserved in all evaluated species, but P56 and H57 can be in some cases replaced by other residues. Noticeably, these two residues are substituted by a Glu and a Ser, respectively, in *Sp*FADS (Figure S1a), an enzyme showing particular requirements regarding the redox state of the flavin for catalysis. Nonetheless, structures for both *Ca*FADS and *Sp*FADS show similar conformation for this consensus region, which corresponds to the β-turn L3n (residues 55–57) and the helix α2n (residues 58–62) in *Ca*FADS (Figure 1). L98, at L6n (residues 93–102), is not situated in any consensus region, but conservative substitutions of hydrophobic nature are usually found in this position (Figure S1a) [21]. Noticeably, none of these residues are conserved in other members of the NT superfamily different from FADSs (Figure S1b), although some NTs conserve the histidine residue that sits between the prolines in FADSs. Taking into account the homology between the FMNAT module of FADSs with NTs [22,23], these observations suggest that these residues must be somehow involved in the flavinic substrate, specific for FADSs, binding.

### 2.2. Mutations at P56, P58 and L98 Produce Subtle Changes in the Environment of Aromatic Residues

Expression levels of *Ca*FADS variants in *Escherichia coli* were similar to those for the wild-type WT, and all of them were purified to homogeneity with a yield of 10–17 mg of protein per g of wet cells mass. As with the WT enzyme, all variants showed a unique absorption maximum at 279 nm in the UV–visible spectra [24]. As expected, variants replaced by tryptophan have slightly greater extinction coefficients than the WT (Table S1). Nonetheless, the increases in ε^279^ observed for the rest of the variants suggest that mutations somehow influence the electronic environment of some aromatic residues. Far-UV CD spectra of *Ca*FADS variants resembled that of the WT (Figure S2a), suggesting that mutations do not impact secondary structure. Nonetheless, a slight emphasis on the 207 nm minimum is observed in the P56W variant, suggesting minor effects in secondary structure elements probably related to proline removal enhancing local flexibility. Some of the mutations produced an important impact in the magnitude of the near-UV circular dichroism (CD) spectra (Figure S2b). This was expected for variants introducing tryptophan residues, but changes in those in which the number of aromatic amino acids was not modified further support that mutations alter the environment of some aromatic residues. These observations agree with changes observed in extinction coefficients (Table S1). Note that, again, the P56W variant exhibits the most significant changes, with the 284 nm minimum becoming a shoulder. Collectively, these data suggest that the mutations do not compromise the overall *Ca*FADS folding—they only introduce small and local conformational changes in the environment of some aromatic side chains. 

### 2.3. P56, P58 and L98 Contribute to Maintain the FMNAT and FADpp Activities of CaFADS and to Modulate Its RFK Activity

**Figure 2:**
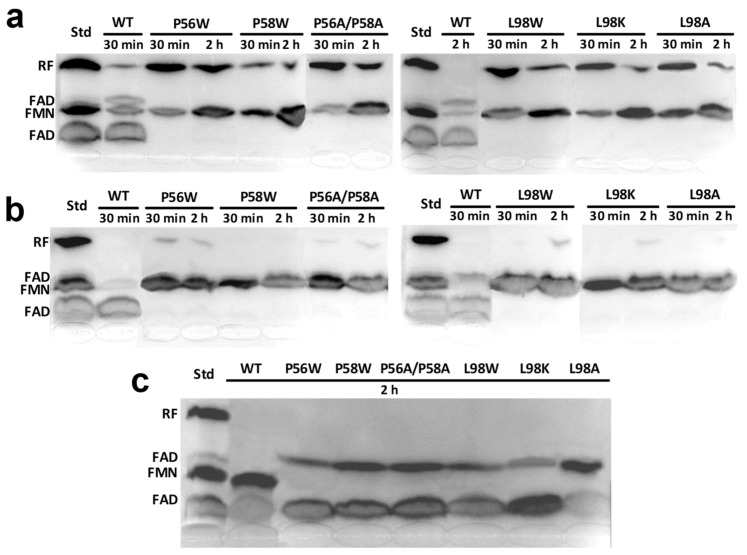
Thin-layer chromatography (TLC) resolution of the products of the transformation of (**a**) Riboflavin (RF), (**b**) FMN and (**c**) FAD by the *Ca*FADS variants after the incubation of reaction mixtures for 30′ and/or 2 h at 25 °C. Reaction mixtures contained 200 nM of enzyme, 50 μM of flavin and 0.2 mM of ATP (in **a**,**b**) or PPi (in **c**) in 20 mM 1,4-Piperazinediethanesulfonic acid (PIPES), 10 mM MgCl_2_, pH 7.0. Standard markers contain 50 μM of each of the flavins (RF, FMN and FAD) incubated in similar conditions.

RFK, FMNAT, and FADpp activities of the *Ca*FADS variants were first qualitatively evaluated by analysis of the reaction products after the incubation of reaction mixtures for 30 min and 2 h (Figure 2). All variants kept the RFK activity, but in general changes in the amount of RF transformed in the reaction times were observed when compared with the WT (Figure 2a). Noticeably, none of the variants was neither capable of catalyzing the transformation of FMN into FAD (Figure 2b) nor of FAD into FMN (Figure 2c). Thus, substitutions at P56, P58, and L98 override the FMNAT and FADpp activities, and modulate the RFK activity. Since the CD spectra of the variants discarded protein unfolding, these mutations must either alter formation of the catalytic complex or prevent the binding of substrates at the FMNAT site. 

**Figure 3:**
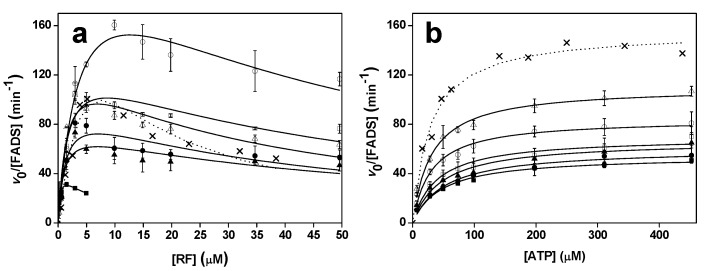
Michaelis–Menten profiles for the RFK activity of WT (cross, fitting in dotted line), P56W (filled square), P58W (filled circle), P56A/P58A (filled triangle), L98W (open square), L98K (open circle) and L98A (open triangle) *Ca*FADS variants (**a**) as a function of the RF concentration at saturating ATP (~400 µM) and (**b**) as a function of ATP at RF concentrations producing ~80% of the maximum activity experimentally observed in (**a**). Reactions were followed in 20 mM PIPES, 0.8 mM MgCl_2_, pH 7.0, at 25 °C.

The kinetic parameters for the RFK activity of the different *Ca*FADS variants were determined by quantifying product formation on the conversion of RF into FMN as a function of time and at different concentrations of substrates. Again, and contrary to the WT, chromatographic profiles fail to detect FAD in these reaction mixtures, confirming the lack of FMNAT activity in these *Ca*FADS variants. All variants showed inhibition of the RFK activity when varying RF concentration (Figure 3a). Such behavior is a species-specific trait of *Ca*FADS that has been associated with an excess of RF substrate and/or FMN and ATP products forming dead-end complexes that prevent the reaction to take place [13,18,25]. Fitting the data to the equation that describes a substrate inhibition mechanism provides lower *k*_cat_ and *K*_m_^RF^ values for the variants than those of the WT *Ca*FADS (Table 1). The impact was greater in the *K*_m_^RF^ value that decreases ~10-fold for most of the variants and up to 30-fold for P56W. Interestingly, with the only exception of P56W, all the variants showed higher *K*_i_ values (up to 10-fold), suggesting reduced affinity for the substrate/product inhibitor. 

Profiles obtained for the RFK activity of the variants on the ATP concentration fitted to the Michaelis–Menten model, as did the WT (Figure 3b). In this case, the variants also showed slightly lower catalytic efficiencies for the ATP substrate, due to the up to 2.5-fold lower *k*_cat_ and the up to 1.8-fold increase in *K*_m_^ATP^, when compared to WT *Ca*FADS (Table 1).

These data indicate, first, that the introduced mutations at P56, P58 and L98 residues abolish the *Ca*FADS FMNAT activity and, second, that the conformation of the FMNAT substrates binding site cavity is somehow related to the binding/exit of substrates/products at the RFK site of the enzyme. 

### 2.4. P56, P58 and L98 Contribute to the Binding of Flavinic Substrates/Products at the FMNAT Site of CaFADS

Since the substitutions studied here nullified the *Ca*FADS ability to transform FMN into FAD, we aimed to assess whether this was a consequence of substrates unable to bind at the FMNAT active site or the interaction happening in an unproductive conformation. We first used differential spectroscopy to qualitatively evaluate the impact of the mutations in the flavin:*Ca*FADS interaction. Taking advantage of the fact that in the absence of adenine nucleotide, both FMN and FAD exclusively bind to the FMNAT cavity [24], we were able to evaluate the impact of the mutations in the binding of flavins at the FMNAT site. All the variants produced difference spectra after incubation with FMN. However, they showed differences, mainly in magnitude, with regard to the WT, indicating that the mutations alter the FMN isoalloxazine electronic environment within the FMNAT site (Figure S3). The magnitude of the difference spectra for P56W, the double P56A/P58A, L98K and particularly L98W variants were significantly lower when compared to the WT protein. Moreover, the FMN concentrations required for achieving the maxima difference spectra signals were significantly higher for variants P56W, P58W and L98W. On the contrary, the titration of *Ca*FADS variants with FAD hardly produced difference spectra, the Δε values observed being negligible (Figure S4), indicating that all the introduced mutations have an important deleterious effect in the placement of the FAD isoalloxazine ring inside its binding cavity. 

**Figure 4:**
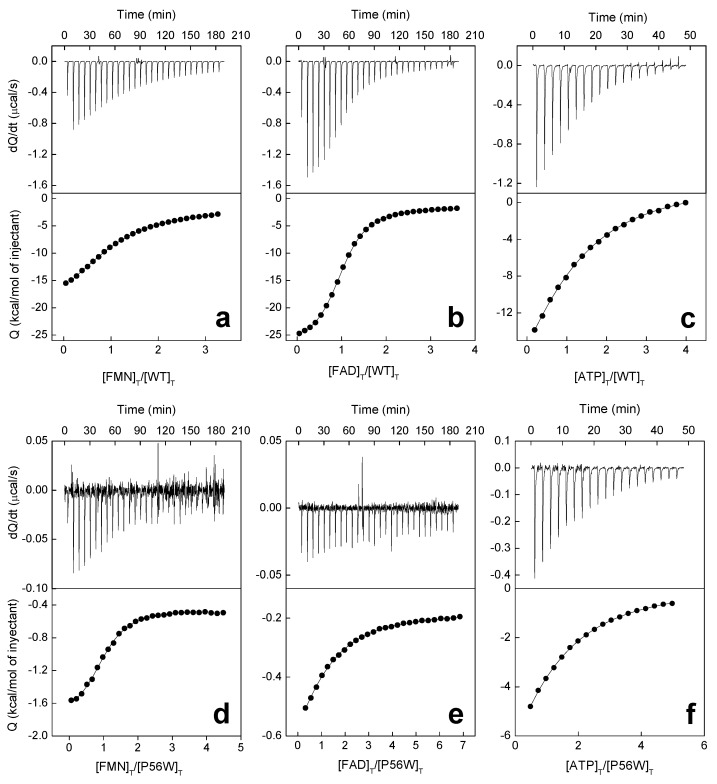
Thermogram (upper panels) and binding isotherms with integrated heat (lower panels) for the titration with FMN (**a**,**d**), FAD (**b**,**e**) and ATP (**c**,**f**) of WT (**a**–**c**) and P58W (**d**–**f**) *Ca*FADS variants in 20 mM PIPES, pH 7.0. Titrations were performed with 10 mM MgCl_2_ for FMN and FAD and in its absence for ATP.

**Figure 5:**
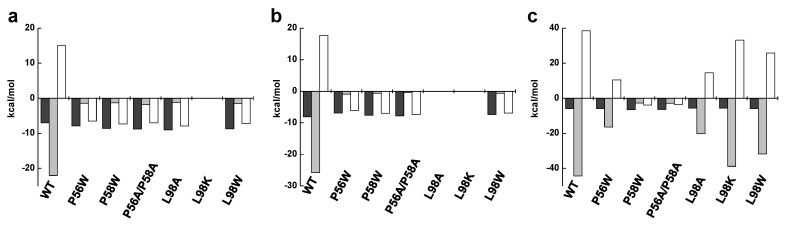
Thermodynamic dissections for the interaction of *Ca*FADS variants with (**a**) FMN, (**b**) FAD and (**c**) ATP. The Gibbs energy (Δ*G*), enthalpy (Δ*H*), and entropy (−TΔ*S*) contributions to the binding are represented in dark grey, light grey and white bars, respectively. Experiments were carried out at 25 °C in 20 mM PIPES, pH 7.0, for ATP, containing 10 mM MgCl_2_ in addition for FMN and FAD titrations. SD values in Δ*G,* Δ*H* and −TΔ*S* are in general ±1 kcal/mol for FMN and FAD binding, and they can considerably increase for ATP binding (Table S2).

We then used isothermal titration calorimetry (ITC) to quantitatively evaluate the effect of the mutations on ligand binding. Under our assay conditions, a single binding site, situated at the FMNAT cavity, has been reported for the titration of WT *Ca*FADS with either FMN or FAD by ITC [24,25]. When our variants were similarly evaluated, no binding thermograms were detected for the titrations of L98K with neither FMN nor FAD, and of L98A with FAD. The rest of the variants kept the same stoichiometry as the WT—one binding site (Figure 4). Derived dissociation constants, *K*_d_, from these experiments indicated that WT *Ca*FADS binds FAD stronger than FMN (1.1 µM and 7.1 µM, respectively) (Table 2). On the contrary, mutations studied here increased the FMNAT module affinity for FMN (with *K*_d_^FMN^ values up to 4-fold lower for P56W and more than 10-fold lower for the rest of the variants) and decreased the protein affinity for FAD (*K*_d_^FAD^ values increased by at least 2-fold, and up to 6 for P56W) (Figure 4a,b, Table 2). The different, and apparently opposite, effects of mutations in FMN and FAD binding has to be understood in the context of FAD being formed by a FMN moiety plus an adenine monophosphate (AMP) moiety that must bind in the ATP binding site of the FMNAT module. Thus, FAD binding requires the simultaneous placement of its flavin and adenine moieties at their respective cavities. The bipartite binding required for FAD would be beyond its reduced ability, when compare to FMN, to fit in a distorted cavity. In fact, contrary to the WT [24], variants exhibited a considerably low enthalpic contribution for FMN and FAD binding, while a favorable entropic contribution became the driving force (Figure 5a,b, Table S2). These observations confirmed that the introduced mutations alter not only the FAD but also the FMN binding modes at the FMNAT site of *Ca*FADS. 

ITC was also used to evaluate ATP binding. In this case, titrations were carried out in the absence of MgCl_2_, since under these conditions, the binding of ATP only takes place at the *Ca*FADS FMNAT module (cation induces a second binding site at the RFK module) [24]. All variants kept the 1:1 binding stoichiometry reported for WT *Ca*FADS in these conditions, but two trends in behavior regarding thermodynamic parameters were observed. Substitutions at L98 and the replacement of P56 by Trp only mildly modulated the parameters for ATP binding when compared to the WT, exhibiting similar thermodynamic profiles and only slightly decreasing the affinity for the nucleotide (Figure 4c, Table 2 and Table S2). On the contrary, the P58W and P56A/P58A variants exhibited lower *K*_d_^ATP^ values and altered the thermodynamic profile for ATP binding, having a negative impact on the enthalpic contribution and converting the entropic one into slightly favorable (Figure 5c and Table S2). According to these results, mutations introduced at P58 compromise ATP binding at the FMNAT module of *Ca*FADS more so than those at P56, while residue L98 hardly influences the nucleotide interaction (in agreement with its situation far from the ATP binding site).

## 3. Discussion

All substitutions introduced here at P56, P58 and L98 in *Ca*FADS override the enzyme ability to catalyze the FMNAT and FADpp activities occurring at the FMNAT module (Figure 2b,c). Nonetheless, all variants bind ATP, and only the L98K and L98A mutations totally prevent the binding of FMN and/or FAD at this module (Figure 4, Table 2). However, thermodynamic profiles for ligand binding indicate that most mutations remarkably impact the FMN, FAD and ATP binding mode (Figure 5a,b, Table S2). In agreement, difference spectra for flavins binding indicate subtle changes in the environment of the flavinic ring of FMN among variants, and the lack of accommodation of the flavinic ring of FAD within its cavity for all of them (Figures S3 and S4). Therefore, mutations at P56, P58 and L98 negatively influence the ability of *Ca*FADS to accommodate the isoalloxazine ring of flavinic substrates within its binding site at the FMNAT module. 

**Figure 6:**
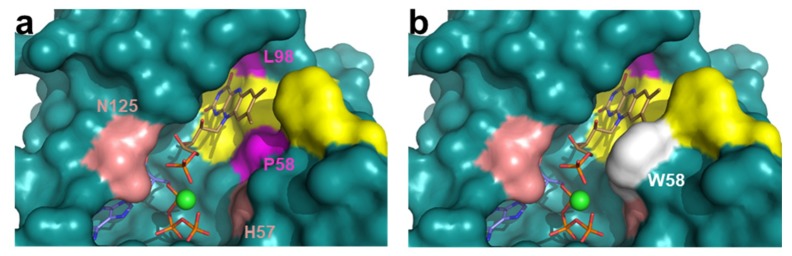
Molecular surface of the active site of the FMNAT module of *Ca*FADS (**a**) in WT and (**b**) in a P56W in silico model with the substituted tryptophan in white. Surfaces of residues F54, F62, F93, Y106 and F128 coating the isoalloxazine binding site are shown in yellow. Substrates are docked according to [20]. Rest of color codes as in Figure 1.

Docking models for the binding of the FMN and ATP:Mg^2+^ substrates at the FMNAT active site in *Ca*FADS [20] situate the isoalloxazine ring in a hydrophobic pocket formed by F54, P58, F62, F93, L98, Y106 and F128, but only the side chains of F54, F62, F93 and Y106 are expected to directly contact the isoalloxazine (Figure 1c and Figure 6). P56, despite not being part of the cavity, forms together with P58 and H57, a highly conserved motive (Figure S1). This motive contributes to the side chain of this histidine approaching the phosphates of substrates from the opposite site of residue N125—the amide group of which has been shown to be key to providing a hydrogen bond to position the phosphates [12,20] (Figure 1b and Figure 6a). We observe different impacts in flavin binding and in environments of aromatic residues as a function of both the modified residue and the nature of the substitution (Figure 5a,b, Figures S2b, S3 and S4, Table 2 and Table S2), in turn suggesting the conformational rearrangements of L3n, α2n and/or L6n, and/or of their environment (Figure 1c). 

P56 and P58 at L3n (residues 55–57) and α2n (residues 58–62), respectively, of the FMNAT module (Figure 1c), appear to confer rigidity to these structural elements at the entrance of the isoalloxazine cavity. Their substitution, particularly in the P56A/P58A variant, must favor flexibility in L3n and α2n (in agreement with the changes in near-UV CD spectra (Figure S1b)), which also contain H57 and F62 (putatively involved in phosphates and isoalloxazine interaction, respectively). The replacement of P58 by the bulky tryptophan will in addition presumably reduce the size of the isoalloxazine entrance and binding sites (Figure 6). Altogether, changes in these structural elements can explain why the P56 and P58 mutations considerably alter FMN and FAD thermodynamic contributions to the binding (Figure 5a,b, Table S3), as well as the protein capability to internalize their isoalloxazine rings, particularly the one of the large molecule of FAD (Figures S3 and S4). Moreover, they can similarly explain why substitutions at P56 and, particularly, P58 modulate the thermodynamic contributions to ATP binding (Figure 5c, Table 2 and Table S2), suggesting that these Pro residues confer a particular conformation to the L3n–α2n motif that contributes to H57 approaching the active site crevice. In this way, H57 can contribute to stabilize the negative charge of phosphates of ATP:Mg^2+^ and FMN at the active site during catalysis.

Our data also show a drastic impact in flavins binding when substituting L98—the replacement of which by Lys and Ala produces variants unable to interact with FMN and FAD (Table 2). Noticeably, in the WT enzyme, the L98 side chain, at L6n, points to the opposite side of the isoalloxazine cavity. Thus, it sits among L1n (residues 6–47), L6n (residues 93–102) and α4n (residues 102–111), surrounded by T6, I92, E97, F109 and L110 (Figure S5). The introduced changes in charge, hydrophobicity and/or size will surely change the conformation in this region. This in turn can produce conformational reorganizations in L1n and L6n loops, as predicted from the changes observed in near-UV CD spectra (Figure S5). Induced changes, particularly in L6n, must be critical to shape the isoalloxazine cavity. Therefore, the nature of the P56, P58 and/or L98 side chains in *Ca*FADS is important to shape a cavity able to internalize the isoalloxazine moiety of the flavinic substrates at its FMNAT site. The fact that the FMNAT activity is not observed even for variants apparently able to internalize the isoalloxazine of FMN (Figure S3) indicates that the nature of these residues is also key to achieving an adequate geometry at the active site for catalysis. In *Ca*FADS, the adenylylation of FMN occurs via a direct nucleophilic attack by the 5’-P of FMN on the α-P of ATP, as also described for other members of the NT superfamily [12,26]. This mechanism requires the reacting phosphates of substrates to get close enough for the nucleophilic attack to take place. The structure of *Ca*FADS reveals an open FMNAT active site [3], where the competent binding of substrates must be carefully driven by residues at their binding cavities. In this way, H28 and H31 are key residues that accommodate the adenine moiety of ATP in such a way that its phosphates are addressed towards the active site, where N125 has been shown to be directly implicated in its stabilization to ensure approaching the FMN phosphate [12,20]. Here, we similarly show how the properties of the isoalloxazine binding cavity (to which P56, P58 and L98 contribute) are key to bind the FMN in a conformation that favors its phosphate group to situate at the active site. Thus, the internalization of the isoalloxazine ring and the formation of the competent complex for catalysis at the FMANT site of *Ca*FADS are highly sensitive to subtle changes in the conformation of the isoalloxazine binding cavity, indicating that there is fine regulation of the binding of the flavin substrate for efficient FMNAT catalysis. Such an idea is in line with former studies showing that some FADSs are only able to internalize and, as a consequence, to transform flavins at their FMNAT site when they are in their reduced state [17], while in others, the transformation of both oxidized or reduced flavins occurs with different catalytic efficiencies [16].

**Figure 7:**
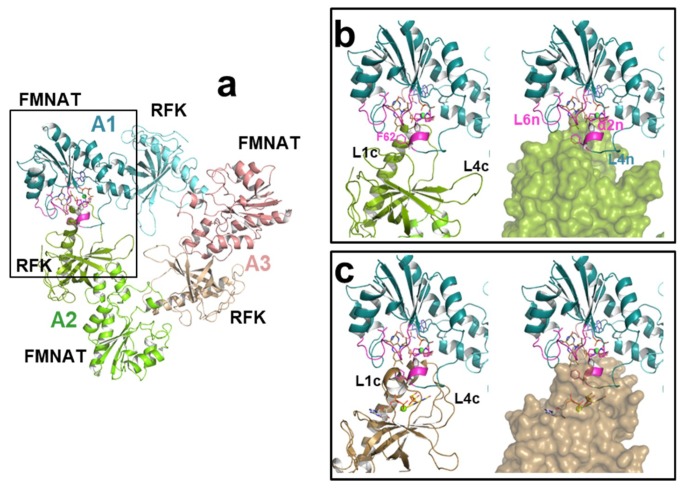
Quaternary structure assembly of *Ca*FADS. (**a**) Cartoon representation of a trimer of the dimer-of-trimers assembly reported for *Ca*FADS. Docked substrates of the FMNAT activity are shown in protomer A1. Cartoon models of the head-to-tail interface of the FMNAT module of protomer A1 (blue) with (**b**) the open conformation of the RFK module (green) in protomer A2 (PDB code: 2x0k) and (**c**) when replacing the conformation of the RFK module in protomer A1 by the closed one (brown) elicited on the binding of the products of the RFK activity (PDB code: 5a89); RFK modules are shown as cartoon and surface on left and right panels, respectively. F62 at the interface between head-to-tail modules is shown as magenta sticks. In (**c**), FMN and ADP products of the RFK activity are shown in CPK sticks with C in yellow and grey, respectively. Other color codes as in Figure 1 and Figure 2.

Furthermore, the main effect of the mutations studied here occurs in the binding of substrates and activity at the FMNAT site, and they also modulate the kinetic parameters for the RFK activity taking place at the RFK C-terminal module of the protein (Figure 2a and Figure 3, Table 1). Such an observation agrees with previous reports indicating that during catalysis in *Ca*FADS, (i) the FMNAT and RFK modules influence the catalytic efficiency of each other [13,15] and (ii) a dimer-of-trimers organization is produced, sitting the RFK module of one protomer and the FMNAT module of the contiguous protomer within each trimer in a head-to-tail organization (Figure 7a) [3,14]. Structural analyses of the binding of ligands to the RFK and FMNAT sites (by using both X-ray diffraction crystallography and docking/MD simulations) have shown large conformational changes in several loops of the RFK module (particularly L1c and L4c) and the relative pivoting of the head-to-tail RFK and FMNAT modules [4,20]. Noticeably, in this organization, the open and free binding site at the RFK module of one protomer is in part covered by α2n and L4n of the contiguous protomer [4] (Figure 7b). Thus, when, on ligands binding at the RFK site, L1c and L4c of the RFK module close its active site cavity, the FMNAT region formed by L3n (containing P56), α2n (containing P58) and L4n of the contiguous protomer need to be displaced, envisaging the approach of L1c towards L6n (containing L98) (compare molecular surfaces in Figure 7b,c) [4]. Such observations agree with P56, P58 and L98 substitutions modulating ligand binding, kinetic and substrate inhibition parameters at the RFK site (Figure 3 and Table 1), effects that can only be explain within the head-to-tail organization. Since, as mentioned above, these substitutions influence conformation at the FMNAT module (Figure S2b), this study further contributes to identifying L3n, α2n and L6n as those regions where the pivoting of the RFK module on the FMNAT one must be produced for substrates access to the RFK active site.

In conclusion P56, P58 and L98 contribute to shape the conformation of the binding site for the flavins at the FMNAT site of *Ca*FADS. The conformation of the isoalloxazine binding cavity in the WT protein appears particularly designed and tuned for flavin internalization, a feature that in turns appears critical to orientate the reactive atoms of the FMN and ATP:Mg^2+^ (or FAD and PPi) substrates in a competent geometry for the FMNAT (or FADpp) catalysis to take place. Our data suggest the contributions of (i) P56 and P58 to reduce the flexibility of L3n and α2n motives, and of (ii) L98 to shape the cavity through the conformation of L6n as key features for FMNAT catalysis to occur in *Ca*FADS. In addition, they also provide further evidence of the structural crosstalk of different protomers within a quaternary *Ca*FADS assembly, not reported so far for any other FADS species, to regulate the RFK catalysis of this enzyme. Finally, our observations further contribute to the understanding of the mechanisms of substrate binding and catalysis in a bacterial protein family considered as an antimicrobial target, particularly pointing to the isoalloxazine binding pocket as a good receptor site for such compounds to prevent the FMNAT activity. 

## 4. Materials and Methods 

### 4.1. Biological Material

The P56W, P58W, L98A, L98K, L98W and P56A/P58A mutations were introduced by site-directed mutagenesis on the pET28a-*Ca*FADS plasmid by Mutagenex^®^ (this company also sequenced the resulting mutated plasmids to confirm they contained the desired mutations). The mutated pET28a-*Ca*FADS plasmids were used to transform BL21(DE3) *E. coli* cells. Transformants were grown as previously described and *Ca*FADS variants were purified following the same protocol as for the WT enzyme [15]. Protein purity was assessed by SDS-PAGE. Purified samples were dialyzed in 20 mM PIPES, pH 7.0, and storage at −20 °C until used. 

### 4.2. Spectroscopic Analysis

Spectroscopic measurements were carried out following previously established conditions and protocols [12,25]. UV–visible absorption spectra were recorded in a Cary 100 spectrophotometer (Agilent Technologies, Santa Clara, CA, USA). Experimental extinction coefficients at 279 nm (ε^279^) for each variant were determined based on the Gill and von Hippel method [27]. In short, the UV–visible absorbance spectrum of the protein was recorded. Then samples were denatured with guanidinium hydrochloride (6 M in 20 mM sodium phosphate, pH 6.5). Then, the absorbance spectrum of the denatured sample was recorded and its concentration was calculated considering the theoretical extinction coefficient determined using the amino acid sequence. This concentration, taking into account the protein dilution in the denaturation process, was then used to determine ε^279^ at the protein maximum by using the native protein spectrum before denaturation. Difference spectroscopy measurements were carried out in 20 mM PIPES, 10 mM MgCl_2_, pH 7.0, with saturating concentrations of ligands. Circular dichroism (CD) spectra were recorded using a Chirascan spectropolarimeter (Appl. Phot. Ltd., Leatherhead, Surrey, UK) at 25 °C. Samples containing 5 µM *Ca*FADS in 5 mM PIPES, 10 mM MgCl_2_, pH 7.0 and 20 µM *Ca*FADS in 20 mM PIPES, 10 mM MgCl_2_, pH 7.0, were used in the far-UV (cuvette path length, 0.1 cm) or near-UV CD (path length, 0.4 cm), respectively.

### 4.3. Qualitative Detection of RFK, FMNAT and FADpp Activities

RFK and FMNAT activities were qualitatively assayed by the incubation of enzymes with substrates and separation of flavins (substrates and products) from reaction mixtures using TLC on Silica Gel SIL-G-25 plates (20 × 20 cm, thickness 0.25 mm) as previously described [12,21]. The reaction mixtures, containing 50 μM RF/FMN (or FAD), 0.2 mM ATP (or PPi) and ~200 nM of *Ca*FADS in 20 mM PIPES, 10 mM MgCl_2_, pH 7.0 (conditions that allow the three reactions catalyzed by WT *Ca*FADS to take place [24]), were incubated for 30 min and 2 h at 37 °C. Reactions were stopped by boiling the preparations for 5 min. Flavin spots were visualized under UV light.

### 4.4. Quantitative Determination of Steady-State Kinetics Parameters for the RFK Activity

The *Ca*FADS RFK activity was measured at 25 °C in 500 µL of 20 mM PIPES, 0.8 mM MgCl_2_, pH 7.0, containing 0.5–45 µM RF and 10–450 µM ATP. Reactions were initiated by the addition of ~20 nM of enzyme. After 1 min incubation at 25 °C, the reactions were stopped by boiling the mixtures for 5 min. The composition of flavins in the supernatant was determined using an Alliance HPLC system (Waters, Milford, MA, USA) equipped with a 2707 autosampler and a HSST3 column (4.6 × 150 mm, 3.5 µm, Waters) preceded by a pre-column (4.6 × 20 mm, 3.5 µm, Waters). The chromatography was developed at 1 mL/min with a 6 min isocratic program of methanol 40% (*v*/*v*) in 5 mM ammonium acetate (pH 6.0). Detection of flavins was carried out using a 2475 Multi λ fluorescence detector (Waters), excitation wavelength of 470 nm and emission wavelength of 530 nm. Under these conditions, the retention times for the flavins were 2.3 min for FAD, 3.4 min for FMN, and 5.5 min for RF. The flavinic products of the reaction were identified by their retention times and quantified through their corresponding standard curves obtained under the same conditions. Controls containing free flavins in the absence of the enzyme were treated similarly to reaction samples to discard flavin degradation during RFK activity determination. The kinetic data obtained for one substrate at saturating concentrations of the second one (as nmol of flavin transformed per min) were interpreted using the Michaelis–Menten kinetic model, obtaining *k*_cat_ and *K*_m_ with errors of ±10%. In case of inhibition, the experimental data were fitted to a model describing the substrate inhibition of a bi-substrate mechanism [28]. In these situations, errors in apparent *K*_m_ and *k*_cat_ (^app^*K*_m_ and ^app^*k*_cat_) increased with the inhibition constant (*K*_i_) value getting closer to *K*_m_^S^. Reaction mixtures were evaluated in triplicates, and final results are expressed as the standard error of the fitted value (value ± SE).

### 4.5. Isothermal Titration Calorimetry (ITC)

Measurements were carried out using a VP-ITC microcalorimeter (MicroCal LLC, Northampton, MA, USA) thermostated at 25 °C. Ligand (200 µM FMN or FAD, and 300 µM ATP) and enzymes (~20 µM of *Ca*FADS) were dissolved in 20 mM PIPES, pH 7.0 (in presence of 10 mM MgCl_2_ for the flavins), and degassed prior to titration. Up to 28 injections of 4 µL of ligand were added to the sample cell (1.4109 mL) containing the enzyme and then mixed via the rotating (1000 rpm) stirrer syringe.

The association constant (*K*_a_), the enthalpy change (Δ*H*) and the stoichiometry (N) were obtained through non-linear regression of the experimental data to a home-derived model for one binding site implemented in Origin 7.0 (OriginLab, Northampton, MA, USA) [12,24]. The dissociation constant (*K*_d_), the free energy change (Δ*G*), and the entropy change (Δ*S*) were obtained from basic thermodynamic relationships. Experiments were performed in triplicate. Results are expressed as the standard deviation of the mean (±SD).

## Figures and Tables

**Table 1:** Steady-state kinetic parameters (*n* = 3, value ± SE) for the RFK activity of *Ca*FADS variants. Parameters obtained in 20 mM PIPES, 0.8 mM MgCl_2_, pH 7.0 at 25 °C.

*Ca*FADS	*k*_cat_^2,3^ (min^−1^)	*K*_m_^RF 2,3^ (µM)	*K*_i_^2,3^ (µM)	*k*_cat_/*K*_m_^RF^ (min^−1^·µM^−1^)	*k*_cat_^3,4^ (min^−1^)	*K*_m_^ATP 4^ (µM)	*k*_cat_/*K*_m_^ATP 3,4^ (min^−1^·µM^−1^)
WT ^1^	408 ± 200	12 ± 3	4.9 ± 3.9	35 ± 21	155 ± 5	28 ± 4	5.5 ± 0.8
P56W	45 ± 5	0.4 ± 0.1	6.5 ± 1.9	125 ± 33	54 ± 2	47 ± 6	1.2 ± 0.2
P58W	98 ± 19	1.3 ± 0.7	39 ± 21	78 ± 43	60 ± 2	52 ± 7	1.2 ± 0.2
P56A/P58A	78 ± 12	0.9 ± 0.4	54 ± 29	87 ± 45	67 ± 4	46 ± 9	1.4 ± 0.3
L98A	138 ± 26	1.5 ± 0.7	32 ± 15	94 ± 48	110 ± 3	30 ± 4	3.6 ± 0.5
L98K	248 ± 34	4.0 ± 1.1	41 ± 13	63 ± 19	84 ± 3	30 ± 5	2.8 ± 0.4
L98W	140 ± 21	1.6 ± 0.6	46 ± 20	85 ± 35	70 ± 4	39 ± 8	1.8 ± 0.4

^1^ Data from [13]. ^2^ Parameters obtained at saturating ATP. ^3^ Strong RF substrate inhibition prevents the determination of real parameters and these correspond to apparent ones—^app^*k*_cat_ and ^app^*K*_m_. Estimated errors in ^app^*k*_cat_ and ^app^*K*_m_ can increase up to ±35% due to *K*_i_ values in the range of *K*_m_^RF^ ones. ^4^ Parameter estimated when using RF concentrations producing ~80% of the maximum experimental activity before inhibition is detected according to Figure 3a.

**Table 2:** Dissociation constants (*n* = 3, mean ± SD) as determined by ITC for the binding of FMN, FAD, and ATP to the *Ca*FADS variants. Data obtained at 25 °C in 20 mM PIPES, pH 7.0, at the indicated MgCl_2_ concentrations. (n.d. interaction not detected during titration).

*Ca*FADS	*K*_d_^FMN^ (μM)	*K*_d_^FAD^ (μM)	*K*_d_^ATP^ (μM)
10 mM MgCl_2_	+	+	-
WT	7.1 ± 0.4	1.1 ± 0.1	48 ± 7
P56W	1.7 ± 0.1	8.3 ± 0.8	44 ± 5
P58W	0.50 ± 0.03	2.6 ± 0.2	16 ± 1
P56A/P58A	0.35 ± 0.02	1.9 ± 0.1	21 ± 3
L98A	0.23 ± 0.03	n.d.	7 ± 5
L98K	n.d.	n.d.	82 ± 21
L98W	0.42 ± 0.02	3.8 ± 0.1	47 ± 7

## Supplementary Materials

Supplementary materials can be found at https://www.mdpi.com/1422-0067/20/20/5083/s1.

## Abbreviations

ATP	Adenosine 5′-triphosphate
CD	Circular dichroism
FAD	Flavin adenine dinucleotide, Riboflavin 5′-adenosine diphosphate
FADpp	FAD pyrophosphorylase
FADS	FAD synthase
FMN	Flavin mononucleotide, Riboflavin 5′-phosphate
FMNAT	ATP:FMN adenylyl transferase
HPLC	High performance chromatography
ITC	Isothermal titration calorimetry
MD	Molecular dynamics
NT	Nucleotidyl transferase
PIPES	1,4-Piperazinediethanesulfonic acid
RF	Riboflavin
RFK	ATP:riboflavin kinase
TLC	Thin-layer chromatography
UV	Ultraviolet
WT	Wilde type

## References

[1] Serrano A., Ferreira P., Martínez-Júlvez M., Medina M. (2013). The prokaryotic FAD synthetase family: A potential drug target. Curr. Pharm. Des..

[2] Karthikeyan S., Zhou Q., Mseeh F., Grishin N.V., Osterman A.L., Zhang H. (2003). Crystal structure of human riboflavin kinase reveals a beta barrel fold and a novel active site arch. Structure.

[3] Herguedas B., Martinez-Julvez M., Frago S., Medina M., Hermoso J.A. (2010). Oligomeric state in the crystal structure of modular FAD synthetase provides insights into its sequential catalysis in prokaryotes. J. Mol. Biol..

[4] Herguedas B., Lans I., Sebastián M., Hermoso J.A., Martínez-Júlvez M., Medina M. (2015). Structural insights into the synthesis of FMN in prokaryotic organisms. Acta Cryst. D Biol. Cryst..

[5] Bauer S., Kemter K., Bacher A., Huber R., Fischer M., Steinbacher S. (2003). Crystal structure of *Schizosaccharomyces pombe* riboflavin kinase reveals a novel ATP and riboflavin-binding fold. J. Mol. Biol..

[6] Barile M., Brizio C., Valenti D., De Virgilio C., Passarella S. (2000). The riboflavin/FAD cycle in rat liver mitochondria. Eur. J. Biochem..

[7] Barile M., Giancaspero T.A., Brizio C., Panebianco C., Indiveri C., Galluccio M., Vergani L., Eberini I., Gianazza E. (2013). Biosynthesis of flavin cofactors in man: Implications in health and disease. Curr. Pharm. Des..

[8] Barile M., Giancaspero T.A., Leone P., Galluccio M., Indiveri C. (2016). Riboflavin transport and metabolism in humans. J. Inherit. Metab. Dis..

[9] Leulliot N., Blondeau K., Keller J., Ulryck N., Quevillon-Cheruel S., van Tilbeurgh H. (2010). Crystal structure of yeast FAD synthetase (Fad1) in complex with FAD. J. Mol. Biol..

[10] Sebastián M., Anoz-Carbonell E., Gracia B., Cossio P., Aínsa J.A., Lans I., Medina M. (2018). Discovery of antimicrobial compounds targeting bacterial type FAD synthetases. J. Enzyme Inhib. Med. Chem..

[11] Efimov I., Kuusk V., Zhang X., McIntire W.S. (1998). Proposed steady-state kinetic mechanism for *Corynebacterium ammoniagenes* FAD synthetase produced by *Escherichia coli*. Biochemistry.

[12] Serrano A., Frago S., Velázquez-Campoy A., Medina M. (2012). Role of key residues at the flavin mononucleotide (FMN):adenylyltransferase catalytic site of the bifunctional riboflavin kinase/flavin adenine dinucleotide (FAD) Synthetase from *Corynebacterium ammoniagenes*. Int. J. Mol. Sci..

[13] Serrano A., Sebastian M., Arilla-Luna S., Baquedano S., Pallares M.C., Lostao A., Herguedas B., Velazquez-Campoy A., Martinez-Julvez M., Medina M. (2015). Quaternary organization in a bifunctional prokaryotic FAD synthetase: Involvement of an arginine at its adenylyltransferase module on the riboflavin kinase activity. Biochim. Biophys. Acta.

[14] Marcuello C., Arilla-Luna S., Medina M., Lostao A. (2013). Detection of a quaternary organization into dimer of trimers of *Corynebacterium ammoniagenes* FAD synthetase at the single-molecule level and at the in cell level. Biochim. Biophys. Acta.

[15] Serrano A., Sebastián M., Arilla-Luna S., Baquedano S., Herguedas B., Velázquez-Campoy A., Martínez-Júlvez M., Medina M. (2017). The trimer interface in the quaternary structure of the bifunctional prokaryotic FAD synthetase from *Corynebacterium ammoniagenes*. Sci. Rep..

[16] Sebastián M., Arilla-Luna S., Bellalou J., Yruela I., Medina M. (2019). The Biosynthesis of Flavin Cofactors in *Listeria monocytogenes*. J. Mol. Biol..

[17] Sebastián M., Lira-Navarrete E., Serrano A., Marcuello C., Velázquez-Campoy A., Lostao A., Hurtado-Guerrero R., Medina M., Martínez-Júlvez M. (2017). The FAD synthetase from the human pathogen *Streptococcus pneumoniae*: A bifunctional enzyme exhibiting activity-dependent redox requirements. Sci. Rep..

[18] Sebastián M., Serrano A., Velázquez-Campoy A., Medina M. (2017). Kinetics and thermodynamics of the protein-ligand interactions in the riboflavin kinase activity of the FAD synthetase from *Corynebacterium ammoniagenes*. Sci. Rep..

[19] Matern A., Pedrolli D., Großhennig S., Johansson J., Mack M. (2016). Uptake and Metabolism of Antibiotics Roseoflavin and 8-Demethyl-8-Aminoriboflavin in Riboflavin-Auxotrophic *Listeria monocytogenes*. J. Bacteriol..

[20] Lans I., Seco J., Serrano A., Burbano R., Cossio P., Daza M.C., Medina M. (2018). The Dimer-of-Trimers Assembly Prevents Catalysis at the Transferase Site of Prokaryotic FAD Synthase. Biophys. J..

[21] Frago S., Martínez-Júlvez M., Serrano A., Medina M. (2008). Structural analysis of FAD synthetase from *Corynebacterium ammoniagenes*. BMC Microbiol..

[22] Krupa A., Sandhya K., Srinivasan N., Jonnalagadda S. (2003). A conserved domain in prokaryotic bifunctional FAD synthetases can potentially catalyze nucleotide transfer. Trends Biochem. Sci..

[23] Huerta C., Borek D., Machius M., Grishin N.V., Zhang H. (2009). Structure and mechanism of a eukaryotic FMN adenylyltransferase. J. Mol. Biol..

[24] Frago S., Velázquez-Campoy A., Medina M. (2009). The puzzle of ligand binding to *Corynebacterium ammoniagenes* FAD synthetase. J. Biol. Chem..

[25] Serrano A., Frago S., Herguedas B., Martinez-Julvez M., Velazquez-Campoy A., Medina M. (2013). Key residues at the riboflavin kinase catalytic site of the bifunctional riboflavin kinase/FMN adenylyltransferase from *Corynebacterium ammoniagenes*. Cell Biochem. Biophys..

[26] Olland A.M., Underwood K.W., Czerwinski R.M., Lo M.C., Aulabaugh A., Bard J., Stahl M.L., Somers W.S., Sullivan F.X., Chopra R. (2002). Identification, characterization, and crystal structure of *Bacillus subtilis* nicotinic acid mononucleotide adenylyltransferase. J. Biol. Chem..

[27] Gill S.C., von Hippel P.H. (1989). Calculation of protein extinction coefficients from amino acid sequence data. Anal Biochem..

[28] Leskovac V. (2003). Comprehensive Enzyme Kinetics.

