# Supplementary material for: Specific Features for the Competent Binding of Substrates at the FMN Adenylyltransferase Site of FAD Synthase from Corynebacterium ammoniagenes
Source: Int J Mol Sci. 2019 Oct 14;20(20):5083. doi: 10.3390/ijms20205083 (PMC6829536; doi:10.3390/ijms20205083)
Supplement: Supplementary file 1 [file ijms-20-05083-s001.pdf]

Appendix A. Supplemental Material

Specific features for competent binding of substrates at the FMN adenylyltransferase site of FAD synthase from *Corynebacterium ammoniagenes*

Sonia Arilla-Luna[1,≠], Ana Serrano[1,2,≠] and Milagros Medina[1,*]

[1] Department of Biochemistry and Molecular and Cellular Biology, Faculty of Sciences, and Institute of Biocomputation and Physics of Complex Systems (Joint Units: BIFI-IQFR and GBsC-CSIC), University of Zaragoza, Spain; arilun2@gmail.com; mmedina@unizar.es

[2] Centro de Investigaciones Biológicas, CSIC, Ramiro de Maeztu 9, E-28040 Madrid, Spain; anaserra1979@gmail.com

* Correspondence: mmedina@unizar.es

≠ These two authors have equally contributed to the study and must both be considered as first authors.

Supplementary Figures

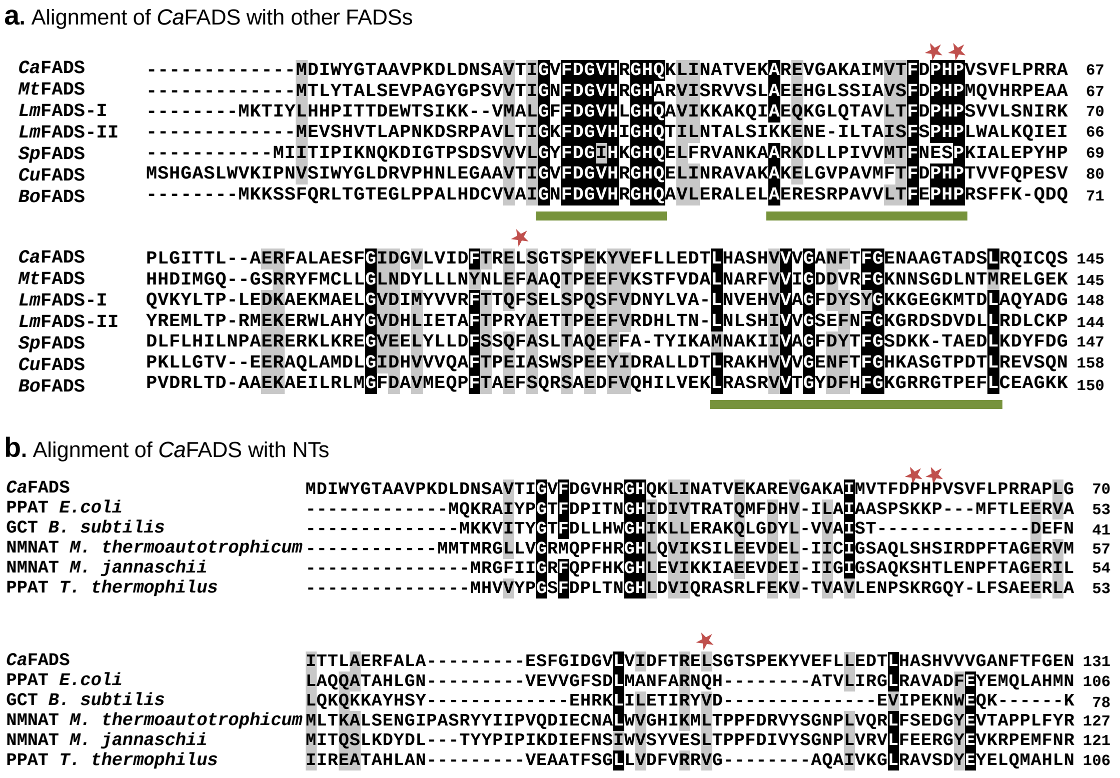

Figure S1. Multiple sequence alignments of (**a**) the FMNAT module in different prokaryotic FADSs (*Mt*FADS, FADS from *Mycobacterium tuberculosis*; *Lm*FADS-I and *Lm*FADS-II, FADSs from *Listeria monocytogenes* type I and type II; *Sp*FADS, FADS from *Streptococcus pneumoniae*; *Cu*FADS, FADS from *Corynebacterium urealyticum*; and *Bo*FADS, FADS from *Brucella ovis*) and (**b**) the FMNAT module of *Ca*FADS with several nucleotidyltransferases (PPAT, phosphopantetheine adenylyltransferase; GCT, CTP:glycerol-3-phosphate citidilyltransferase; NMNAT, nicotinamide mononucleotide adenylyltransferase). Residues shaded in black and grey show respectively ≥80 % of identity and similarity. The highly conserved motifs are underlined in green. Residues here studied are marked with a red star.

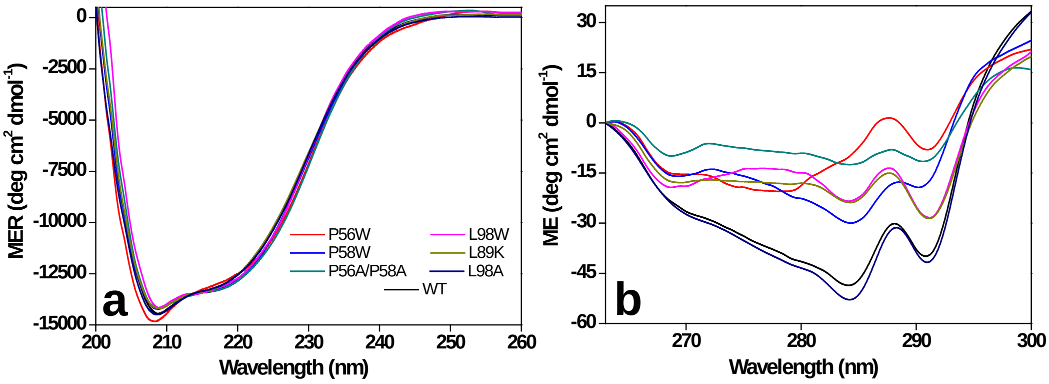

Figure S2. Circular dichroism spectra (molar ellipticity) (**a**) in the far-UV region (*per* residue) and (**b**) in the near-UV region for the different *Ca*FADS variants. Spectra were recorded respectively in 5 mM and 20 mM PIPES, 10 mM $MgCl_2$, pH 7.0 at 25 °C.

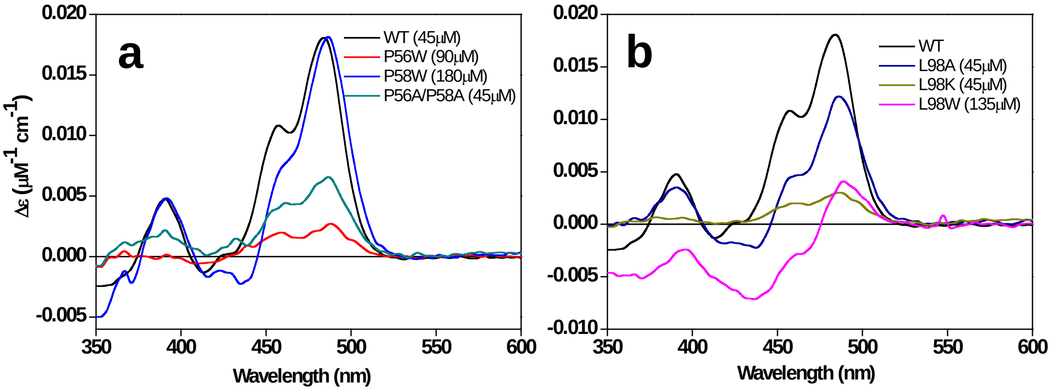

Figure S3. Visible difference spectra elicited upon titration of (**a**) WT, P56 and P58 and, (**b**) WT, and L98 *Ca*FADS variants (4-6 μM) with saturating FMN concentrations (indicated in parenthesis for each variant). Spectra recorded in 20 mM PIPES, 10 mM $MgCl_2$, pH 7.0 at 25 °C.

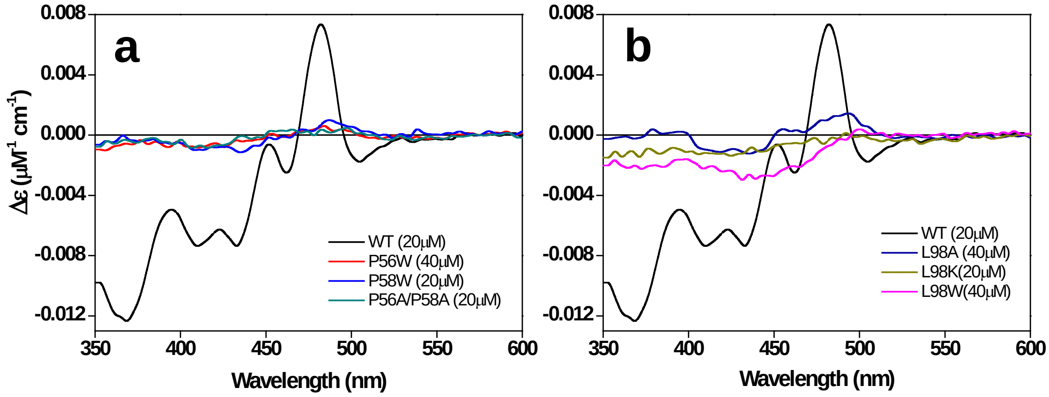

Figure S4. Visible difference spectra elicited upon titration of (**a**) WT, P56 and P58 and, (**b**) WT, L98 *Ca*FADS variants (4-6 μM) with saturating FAD concentrations (indicated in parenthesis for each variant). Spectra recorded in 20 mM PIPES, 10 mM $MgCl_2$, pH 7.0 at 25 °C.

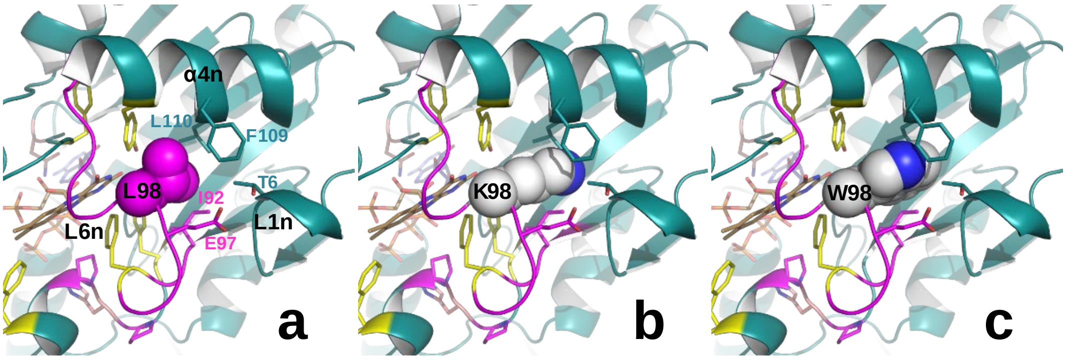

Figure S5. Cartoon detail of the environment of position 98 (shown as spheres) in (**a**) WT *Ca*FADS as well as in (**b**) L98K and (**b**) L98W variants *in silico* models. Mutated K and W residues are CPK coloured with C in white. T6, I92, E97, F109 and L110 side chains surrounding L98 are shown in sticks. Substrates are docked according to [1]. Rest of colour codes as in Figures 1 and 6.

Supplementary Tables

Table S1. Extinction coefficients at 279 nm and 25 °C (n=3, mean ± SD) for the *Ca*FADS variants in 20 mM PIPES, 10 mM $MgCl_2$, pH 7.0.

	ε^{279} in Gdn/HCl[a] ($mM^{-1}cm^{-1}$)	ε^{279} in PIPES ($mM^{-1}cm^{-1}$)	$(\varepsilon^{279}_{PIPES} - \varepsilon^{279}_{Gdn/HCl})/\ \varepsilon^{279}_{PIPES}$[b] (%)
WT	27.4	27.5 ± 0.2	0.4
P56W	33.1	36.8 ± 0.2	10.1
P58W	33.1	36.7 ± 0.1	9.8
P56A/P58A	27.4	30.8 ± 0.1	11.0
L98A	27.4	30.3 ± 0.2	9.6
L98K	27.4	31.5 ± 0.1	13.0
L98W	33.1	34.8 ± 0.4	4.9

[a] Theoretical value based on the amino acid sequence according to [2].

[b] Difference between the theoretical value under denaturing conditions and for the wild-type folded enzyme value is around 0.4 %. For the variants this difference increases up to 10-13 %. The extinction coefficient under denaturing conditions only has into account the number of tryptophan, tyrosine and cysteine (in its reduced state) residues. However, the extinction coefficient under native conditions is influenced by other factors, such as the protein folding around these residues, the buffer (pH and ionic strength), or the particular electronic environment of each residue contributing to it. This is why the calculated extinction coefficient for the native protein does not match with the theoretical value that corresponds to an unfolded protein.

Table S2. Thermodynamic parameters for the interaction of *Ca*FADS variants with FAD, FMN in 20 mM PIPES, 10 mM $MgCl_2$, pH 7.0, and ATP in 20 mM PIPES, pH 7.0. (n=3, mean ± SD).

	Ligand	ΔH (kcal/mol)	ΔG (kcal/mol)	$-T\Delta S$ (kcal/mol)
WT	FMN	-22 ± 1	-7.0 ± 0.1	15 ± 1
	FAD	-26 ± 1	-8.1 ± 0.1	18 ± 1
	ATP	-44 ± 6	-5.9 ± 0.1	39 ± 6
P56W	FMN	-1.4 ± 0.1	-7.9 ± 0.1	-6.5 ± 0.1
	FAD	-0.9± 0.1	-6.9 ± 0.1	-6.1 ± 0.1
	ATP	-16 ± 3	-5.9 ± 0.1	11 ± 2
P58W	FMN	-1.3 ± 0.1	-8.6 ± 0.1	-7.3 ± 0.1
	FAD	-0.56 ± 0.02	-7.6 ± 0.1	-7.0 ± 0.1
	ATP	-2.7 ± 0.1	-6.5 ± 0.1	-3.8 ± 0.1
P56A/P58A	FMN	-1.8 ± 0.1	-8.8 ± 0.1	-7.0 ± 0.1
	FAD	-0.39 ± 0.1	-7.8 ± 0.1	-7.4 ± 0.1
	ATP	-2.8 ± 0.2	-6.4 ± 0.1	-3.6 ± 0.2
L98A	FMN	-1.2 ± 0.1	-9.0 ± 0.1	-7.9 ± 0.1
	FAD	n.d.[a]	n.d.[a]	n.d.[a]
	ATP	-20 ± 3	-5.6 ± 0.1	15 ± 2
L98K	FMN	n.d.[a]	n.d.[a]	n.d.[a]
	FAD	n.d.[a]	n.d.[a]	n.d.[a]
	ATP	-39 ± 19	-5.6 ± 0.2	33 ± 19
L98W	FMN	-1.5 ± 0.1	-8.7 ± 0.1	-7.2 ± 0.1
	FAD	-0.6 ± 0.1	-7.4 ± 0.1	-6.9 ± 0.1
	ATP	-32 ± 6	-5.9 ± 0.1	26 ± 6

[a] n.d. indicates that no interaction profile was detected.

REFERENCES

1. Lans, I., Seco, J., Serrano, A., Burbano, R., Cossio, P., Daza, M.C., and Medina, M. (2018). The Dimer-of-Trimers Assembly Prevents Catalysis at the Transferase Site of Prokaryotic FAD Synthase. Biophys J *115*, 988-995.
2. Gill, S.C., and von Hippel, P.H. (1989). Calculation of protein extinction coefficients from amino acid sequence data. Anal Biochem *182*, 319-326.
